# Management of intra-abdominal hypertension and abdominal compartment syndrome: a review

**DOI:** 10.1186/1752-2897-8-2

**Published:** 2014-02-05

**Authors:** Leanne Hunt, Steve A Frost, Ken Hillman, Phillip J Newton, Patricia M Davidson

**Affiliations:** 1 University of Technology, Sydney & The University of Western Sydney, Locked Bag 1797, Penrith, NSW 2751, Australia; 2 Liverpool Hospital & The University of Western Sydney, Locked Bag 1797, Penrith, NSW 2751, Australia; 3 Liverpool Hospital & The University of New South Wales, Elizabeth St, Liverpool, NSW 2170, Australia; 4 Centre for Cardiovascular and Chronic Care, Faculty of Health, University of Technology Sydney & St Vincent’s & Mater Health Sydney, P.O. Box 123 Broadway, Ultimo, NSW 2007, Australia

**Keywords:** Intra-abdominal pressure, Intra-abdominal hypertension, Abdominal compartment syndrome, Abdominal perfusion pressure

## Abstract

Patients in the intensive care unit (ICU) are at risk of developing of intra abdominal hypertension (IAH) and abdominal compartment syndrome (ACS).

Aim: This review seeks to define IAH and ACS, identify the aetiology and presentation of IAH and ACS, identify IAP measurement techniques, identify current management and discuss the implications of IAH and ACS for nursing practice. A search of the electronic databases was supervised by a health librarian. The electronic data bases Cumulative Index of Nursing and Allied Health Literature (CINAHL); Medline, EMBASE, and the World Wide Web was undertaken from 1996- January 2011 using MeSH and key words which included but not limited to: abdominal compartment syndrome, intra -abdominal hypertension, intra-abdominal pressure in adult populations met the search criteria and were reviewed by three authors using a critical appraisal tool. Data derived from the retrieved material are discussed under the following themes: (1) etiology of intra-abdominal hypertension; (2) strategies for measuring intra-abdominal pressure (3) the manifestation of abdominal compartment syndrome; and (4) the importance of nursing assessment, observation and interventions. Intra-abdominal pressure (IAP) and abdominal compartment syndrome (ACS) have the potential to alter organ perfusion and compromise organ function.

## Background

The importance of the diagnosis and management of intra-abdominal hypertension (IAH) and abdominal compartment syndrome (ACS) is increasingly recognised. These conditions can alter organ perfusion and as a consequence end organ function. Complications resulting from IAH and ACS can be life threatening to critically ill patients [1,2]. Intra abdominal hypertension and ACS have been recognised since the 1800 s [1,2] however, it has only been the past 15 years that the physiological complications of IAH and ACS and the impact these can have on patients has been appreciated. Furthermore, there is limited data published specific to the nursing role in IAH and ACS.

The increase in awareness of IAH and ACS is due to improvements in diagnostic practices and changing treatment paradigms in patients sustaining traumatic injury and those with critical illness [2,3]. Despite the increase in awareness and guideline recommendations, there remains some resistance to adopting regular screening and monitoring practices [4,5]. Spencer et al. [6], in an Australian survey of 582 critical care nurses that the majority (356 or 62.1%) described their knowledge of ACS to be non-existent or limited. Within the same survey it was identified that there is a shortfall in nurses’ knowledge in identifying patients in high risk groups and the clinical manifestations of IAH and ACS. The incidence of IAH in critical care patients is reported to be 50%, of these 50%, 32.1% develop IAH and 4.2% develop ACS within their first day of ICU [7,8]. The pathology is a frequent occurrence in critical care it is essential for nurses to regularly monitor IAP and organ perfusion to predict adverse consequences and be proactive in the management of patients at risk [2,6].

This review seeks to define IAH and ACS, identify the etiology and presentation of IAH and ACS, identify IAP measurement techniques, identify current management and discuss the implications of IAH and ACS for nursing practice.

## Method

An integrative review is a method that permits the inclusion of a range of study designs to provide an inclusive evaluation [9]. This process is particularly informative in intervention development. Following consultation with a health care librarian, the electronic databases CINAHL, Medline, Embase and the Internet were searched databases were searched from 1996 to July 2013. Key word searches of the electronic databases included; abdominal compartment syndrome, abdominal pressure, peritoneal cavity, compartment syndrome, decompression surgery, practice guideline, multiple organ failure, abdominal injury, intensive care, critical illness, risk factors, treatment outcomes, intensive care unit, nursing, nursing care, intra- abdominal hypertension, intra-abdominal pressure, abdomen, critical care, critical illness, wounds and injuries nursing assessment, hypertension. Database searches were limited to the English language and humans. The reference lists of published materials were searched for additional literature. Journals held locally were hand searched for relevant articles. The World Wide Web was searched using Google Scholar and Yahoo search engines for peer reviewed related electronic documents. All abstracts were reviewed for relevance to the aims of the review.

**Figure 1:**
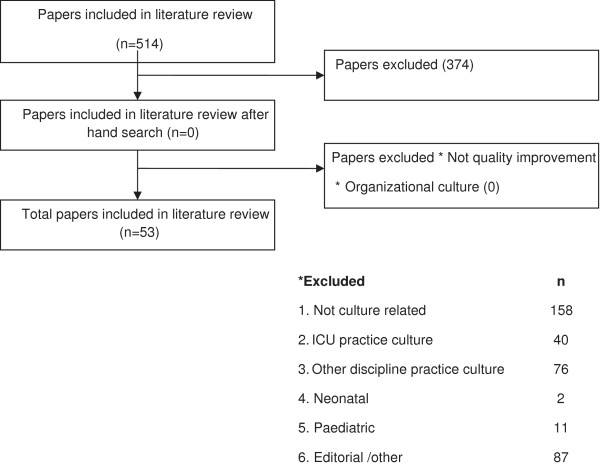
Flowchart of study selection process.

Using the stated search strategy 514 articles were retrieved. Abstracts were reviewed for relevance to the review aims. Sixty five articles provided information describing the nursing role, the description of the assessment process, diagnosis and management of IAH and ACS (Figure 1). The results of the search were analysed by the authors using content analysis driven by the research questions and aims of the study.

A narrative review of the articles is presented and organised into the following themes: (1) Diagnosis of intra abdominal hypertension; (2) etiology of intra-abdominal hypertension; (3) strategies for measuring intra-abdominal pressure; (4) the manifestations of abdominal compartment syndrome; and (5) the importance of nursing assessment, observations and intervention.

## Results

### Diagnosis of intra abdominal hypertension

Intra-abdominal pressure is defined as the pressure created within the abdominal cavity the normal IAP for critically ill adults is 5–7 mmHg [10,11]. Intra-abdominal hypertension is a sustained or repeated IAP > than 12 mmHg [11]. There are various grades of IAH, Grade 1 IAP 12–15 mmHg, Grade 2 IAP 16–20 mmHg, Grade 3 IAP 21–25 mmHg and grade 4 an IAP > 25 mmHg [10-12]. The IAP measurement is completed twice over a period of 1 – 6 hours [13]. If IAP measurements are >12 mmHg but >20 mmHg the WSACS suggest IAP measurements fourth hourly whilst the patient is critically ill, avoid excessive fluid resuscitation and optimize organ perfusion [10,11].

### Etiology of intra abdominal hypertension

There are multiple physiological factors that have the potential to alter an individual’s intra-abdominal pressures (IAP). These factors can be categorised as those that are related to;

1. A decrease in abdominal wall compliance.

2. An increase in intraluminal contents.

3. Capillary leakage or fluid resuscitation see Table 1.

**Table 1:** Physiological factors impacting on intra abdominal pressure (IAH)

Related to diminished abdominal wall compliance	• High BMI
	• Pregnancy
	• Mechanical ventilation
	• The use of PEEP or when auto PEEP is present
	• Basal pneumonia
	• Pneumoperitoneum
	• Abdominal surgery particularly with tight abdominal closures
	• Pneumatic anti shock garments
	• Prone positioning
	• Abdominal wall bleeding or abdominal hematoma
	• Burns with abdominal eschars
Related to increased intra-abdominal contents	• Gastro paresis
	• Gastric distension
	• Ileus
	• Volvulus
	• Bowel pseudo obstruction
	• Abdominal hematoma
	• Intra-abdominal or retroperitoneal hematoma
	• Damage control laparotomy
	• Liver dysfunction with ascites
	• Abdominal infection (peritonitis, pancreatitis)
	• Hemoperitoneum
	• Pneumoperitoneum
	• Major trauma
	• Excessive inflation during laparoscopy
	• Peritoneal dialysis
Related to capillary leak and fluid resuscitation	• Acidosis (pH below 7.2)
	• Hypothermia (core temp below 33°
	• Coagulopathy
	• Multiple transfusions/trauma (>10 units in 24 hours)
	• Sepsis, severe sepsis or bacteraemia
	• Septic shock
	• Massive fluid resuscitation (>5 L colloid or > L crystalloid in 24 hours in the presence of capillary leak and a positive fluid balance)
	• Major burns

Whilst there are no risk prediction models that will assist in identifying IAH or ACS, elevated peak ventilation pressures, decreased urine output, hypothermia, coagulopathy and acidosis have been described in several studies as the key indicators of an increased mortality [14-17]. These same studies suggest early recognition and management of hypothermia coagulopathy and acidosis could result in an overall reduction in in mortality [14-17].

### Intra-abdominal pressure measurement

Measurement of IAP is simple, inexpensive, safe and accurate method in determining the presence of IAH. This measurement can guide patient management [2,10,18,19]. The WSACS has recommended the use of a standardised protocol despite this recommendation across centers there is minimal standardisation of the methods of assessment [7]. Techniques are influenced by measurement accuracy and reproducibility, budget constraints for equipment and staff training and ease of use of the chosen method of measurement [7].

Historically physical observation and measurement of abdominal girth were used to determine the presence of IAH. This method of measurement is inaccurate due to a high risk of variability and low inter-rater reliability [19,20]. A range of approaches to measure IAP include intra gastric, intra rectal, inferior vena cava and via a urinary indwelling catheter pressure monitoring systems [18,20].

The WSACS advocates the use of the modified Kron technique as the gold standard of IAP measurement [2,10,11]. The Kron method assesses the IAP via bladder pressure measurement using a maximum instillation of 25 ml of sterile saline [11]. The measurement is taken;

1. With the transducer zeroed and positioned in line with the iliac crest and mid-axillar.

2. With the patient in a supine position.

3. At end-expiration.

4. With an instillation volume of no greater than 25 mL of saline (for bladder technique).

5. 30–60 seconds after instillation to allow for bladder detrusor muscle relaxation (for bladder technique) [1,10-12,16,20-22].

The reliability of the intermittent measurement guidelines set down by WSACS has been challenged [22,23]. More recently, the technology of continuous IAP monitoring has been suggested to be superior to the intermittent technique [23]. The continuous method allows for continuous analysis of the IAP via the bladder and eliminates the risk of missing elevations in IAP due to timing, which can occur with intermittent techniques [10,13,24]. Continuous methods have been used via the gastric route and invasive direct measurements, but measurements using these techniques showed poor reproducibility [23,25]. However a recent study showed comparable results between the traditional Kron technique and continuous direct intra-abdominal technique [26]. The agreement of continuous bladder IAP measurements to the current gold standard of intermittent measurements is reliable [24,27,28]. The continuous IAP measurement technique requires insertion of the more expensive three way catheter, which could be the cause of its limited use [24,25].

There is also a range of opinions regarding the volume of fluid required to be instilled into the bladder to format an accurate pressure reading. Volumes as low as 2 ml have been used to measure IAP and results are comparable to the use of 25 ml of normal saline [29]. Fluid volumes above 25 ml have the potential to mislead treatment due to overestimation of the IAP [24,29,30]. Current guidelines suggest a maximum volume of 25 ml of fluid be instilled into the bladder for IAP measurement [11].

There are a select few patients in whom IAP measurement via the direct bladder method is not feasible. These include patients with a ruptured bladder, intra bladder hematoma, neurogenic bladder, recent bladder surgery and uro-genital anomalies [31-33]. As suggested by Malbrain et al. [11], the best technique to measure IAP is one critical care nurses will use in their nursing practice [19].

### What is abdominal compartment syndrome

Abdominal compartment syndrome is defined as a sustained IAP greater than 20 mmHg with a new organ dysfunction or failure regardless of abdominal perfusion pressure (APP) [1,2,6,10,12,13,15]. For example, the development of renal failure, respiratory failure or an unexplained metabolic acidosis. The WSACS suggests using these absolute value as a guide when defining ACS recommending that if the patient exhibits signs of new organ dysfunction or failure that this is more clinically significant than an absolute value [10,11].

Abdominal compartment syndrome is further classified into three groups primary, secondary and recurrent ACS.

#### Primary ACS

Primary ACS occurs as a result of injury or disease to the abdominal or pelvic region that frequently requires early radiological or surgical intervention or, conditions that develop post abdominal surgery requiring surgical intervention [2,7,10,11]. Included in primary ACS are patients who are managed non-operatively for organ damage who then go on to develop ACS. This category can include patients with abdominal trauma, abdominal lesions, retroperitoneal hematoma and those associated with damage control during a laparotomy procedure [2,11,12,34].

#### Secondary ACS

Secondary ACS is an often unavoidable progression of the ICU patient’s pathology and refers to conditions that do not originate from the abdominal or pelvic region [11]. Secondary ACS occurs in the absence of any abdominal injury. This can include patients who have sepsis, pancreatitis or have had excessive fluid resuscitation [2,10,13,35].

#### Recurrent ACS

Recurrent ACS is the reoccurrence of ACS after surgical or medical treatment of either primary or secondary IAH or ACS [2,10-12,34].

#### Abdominal perfusion pressure (APP)

The abdominal perfusion pressure (APP) has been identified as an indicator for adequate abdominal perfusion [10,36]. Abdominal perfusion pressure indicates the pressure available for perfusion of intra abdominal organs [10,12]. Abdominal perfusion pressure is calculated using the formula mean arterial pressure MAP – IAP [10,11]. Abdominal perfusion pressure has previously been suggested as a more accurate indicator of IAH severity and indicates the degree of abdominal tissue perfusion [36]. Malbrain and colleagues have also previously recommends that APP should be maintained between 50–60 mmHg for patients with IAH who do not require immediate intervention [8,10,19]. Cheatham et al. and Spencer et al. also suggest that patients with IAH who are unable to maintain an APP above 50 mmHg require surgical intervention [6,36]. More recent studies have suggested that patients with an APP greater than 60 MmHg have shown a reduction in the incidence of renal failure [2,6,10]. Despite these studies the WSACS 2013 consensus management statement could make no recommendations for the use of APP in the resuscitation or management of patients [11].

### Indications for IAP monitoring

There is considerable debate regarding the applicability of absolute IAP ranges in the management of critically ill patients [4,6]. As suggested by multiple authors [4,6,8,10,18], an IAP >20 mmHg can cause significant physiological disturbance in critically ill patients. However, there are also patients with this same elevation in IAP that show no such derangement. Due to differences in clinical presentations there appears to be a lack of clinical awareness hence failure to recognise IAH and ACS [7,14,37]. The WSACS has developed definitive evidence based IAH assessment, IAH and ACS management algorithms and a non-operative management algorithm to improve awareness and management of patients at risk of IAH and ACS [10,11].

Identifying patients at risk is the initial step in the recognition and diagnosis of these pathologies [10]. It is essential that patients are screened for the presence of IAH or ACS upon admission to ICU and additionally in the presence of new or progressive organ failure [2,12,38]. The WSACS suggests assessment for risk factors of IAH and ACS on admission to ICU and for the duration of the patients critical illness [10]. Post assessment if there are two or more risk factors present or there is a new or progressive organ failure then a baseline IAP measurement should be taken then the assessment algorithm should be implemented [10]. If IAH is present medical management should be implemented to reduce IAP, measurements should be taken 4–6 hourly or continuously [10]. For patients with an elevated IAP monitoring should occur throughout the patients critical illness [10].

There are recognised independent risk factors for the development of IAH and ACS [10] (See Table 1). In addition to these independent risk factors IAP monitoring is also suggested for patients with open or blunt abdominal trauma, those who have a high body mass index (BMI), those who sustain burns, or hypotensive for whatever reason, those patients with mesenteric ischemia or patients with an elevated ICP [7,10].

### Implications for nursing practice

In spite of the diverse literature discussing IAH and ACS, there is limited literature specific to the nursing care for patients with IAH or ACS. Patients with IAH or ACS will be most frequently encountered in ICU, high dependency units (HDU), coronary care units (CCU) and emergency departments (ED). Recently, it has been proposed to expand IAP and ACS monitoring beyond traditional critical care areas to enable early detection of the clinical deterioration in in susceptible patients thus improve patient outcomes [32,39].

The complex presentation of patients with IAH or ACS requires an advanced practice nurse’s clinical expertise and vigilant monitoring is essential [6,21]. Advanced practice nurses possess superior assessment and decision making skills, critical thinking and communication expertise that is imperative in an often unpredictable critical care environment [40]. Advanced nursing practice allows expert nurses to demonstrate increased clinical discretion, responsibility and autonomy when recognising, assessing, and managing patients with IAH or ACS [41].

Specific nursing management is focused on assessing organ function, pain management, vital signs, perfusion to the lower extremities, assessment of wound drainage and output, ongoing assessment for reoccurrence of IAH or ACS and provision of support to patients and their families [6,42,43].

#### Organ function

Due to the adverse effects of IAH and ACS on patient morbidity and mortality (See Table 2), there is a need for advanced practice nurses to assess and manage patients using evidence based protocols [38].

**Table 2:** Adverse effects of intra abdominal hypertension (IAH) and abdominal compartment syndrome (ACS)

Cerebral	• An Increase in IAP forces the diaphragm up decreasing intra-thoracic space, increasing the intra-thoracic pressure.
	• Jugular venous pressure elevates.
	• Venous return decreases.
	• Intra cerebral pressure will increase.
	• Cerebral blood flow decreases.
Cardiac function	• An increase in IAP causes increased pressure on the inferior vena cava, intra abdominal circulation and perfusion.
	• Venous return is impaired and peripheral oedema occurs.
	• Increase in central venous pressure.
	• Increased pulmonary artery wedge pressures as the myocardium is placed under an increasing workload.
Respiratory function	• An increased in IAP forces the diaphragm up decreasing intra-thoracic space and restricts respiration.
	• Result in an increase in intra thoracic pressure particularly with mechanically ventilated patients.
	• Left uncorrected will result in a decrease in lung compliance, functional residual capacity a VQ mismatch and hypoxia.
Renal function	• Defined as oliguria and anuria despite aggressive fluid resuscitation.
	• Increase in abdominal pressure decreases renal blood flow coupled with a reduction in cardiac output.
	• The rennin angiotensin system is activated further adding to intra- abdominal pressure and cardiac workload.
Gastrointestinal function	• Increased intra- abdominal pressure results in an increase in vascular resistance and decreased cardiac output.
	• Results in a decrease in tissue perfusion.
	• Ultimately tissue ischemia.
Peripheral perfusion	• Increased intra- abdominal pressure is said to increase femoral venous pressure increase peripheral vascular resistance and reduce femoral artery blood flow by up to 60%.

Patients with ACS are often managed with pharmacological, technical, medical and surgical procedures [11,44,45]. Pharmacological support for patients with IAH or ACS is multi-faceted and entails active and precise fluid resuscitation to maintain adequate circulating volume without fluid overloading, medications to support cardiac output in the event of decompensation and antibiotics to treat infections [6,42,46-48]. In the context of a critical illness, technical support involves ventilator support, continuous renal replacement therapy (CRRT), invasive cardiac monitoring, arterial blood gas interpretation and intervention, blood glucose monitoring and treatment of electrolyte disturbances [6,42,49].

A non- surgical approach is generally used in patients with no abdominal injuries and may involve the insertion of a percutaneous drain for fluid removal [44,48,50,51]. Current guidelines suggest that when IAH or ACS has been established and intra peritoneal fluid has been confirmed percutaneous drainage should be undertaken as it may negate the need for decompressive laparotomy [11]. Other measures endorsed by the WSACS include the judicious use of fluids, endogastric tube insertion, laxative usage, pain relief and muscle relaxants [6,8,11,32,48,52]. Whilst other measures such as CRRT, diuretics and albumin are being used to manage patients the WSACS could make no recommendations regarding their use [11,49]. Another non-surgical approach to prevent and manage IAH and ACS is damage control resuscitation. Damage control resusitation is chacterised by permissive hypotension, limitation of crystalloid infusion and the administration of higher ratios of plasma and platelets to red blood cells [17,53]. The WSACS suggests a higher ratio of plasma and packed red blood cells as opposed to limited or no use [11].

Surgical management involves decompression of the abdomen [17,54]. Decompression occurs in cases of trauma with abdominal injuries or where the patient’s clinical condition continues to deteriorate while using non-surgical management techniques. Decompression is aimed at restoring organ perfusion and ultimately organ function. Early surgical decompression of the abdomen is considered a therapeutic intervention and a definitive treatment for ACS and is performed when ACS is unresponsive to medical treatment options [1,10-12,52]. This is recommended despite reported complications and 50% mortality rates [11,52]. Decompression often results in the abdomen being left open followed by other surgical procedures [6,10,15,32,55-57]. Presumptive decompression should be considered at the time of laparotomy for patients who demonstrate risk factors for ACS [7,58]. After a decompression procedure where the abdomen is left opened there is limited literature guiding definitive abdominal closure. It has been suggested that closure is possible within 5–7 days of decompression if the patient underwent early decompression prior to the development of significant organ injury [7,58]. However, optimal timing of closure is dependent upon normalisation of IAP [6].

Damage control laparotomy for trauma patients is used as a measure to control hemorrhage and restore metabolic function and is supported as a resuscitative procedure by the WSACS [11,17,59]. Current guidelines suggest this method should be used when the patient is physiologically fatigued with the abdomen to remain prophylactically opened to avoid IAH [11].

The role of the nurse is to assess, interpret and titrate therapy according to the patients’ organ function [6,42]. Nursing care of the patient with an open abdomen involves the management of complex wounds, negative pressure systems, assessment of vascular supply to the wound, wound drainage, dressing integrity, patient positioning, and assessing for recurrence of ACS [6,42]. Unless contraindicated, nasogastric feeding should be considered to optimise gastrointestinal function [60,61].

### Implications for further research

The research surrounding the care of the patient with IAP and ACS is limited and hence, further research is required. This research will;

1. Improve the body of knowledge about IAH and ACS within nursing.

2. Provide nurses with the knowledge to identify patients at risk.

3. Improve patient outcomes.

Intra-abdominal hypertension and ACS are potential life threatening conditions to critically ill patients. Critical care nurses have the ability to identify IAH and ACS, implement and evaluate management interventions. Nursing practice should be centered on evidence based practice guidelines [62]. Nurses should provide a standard of care in managing patients who are at risk of IAH and ACS from pre-hospital, emergency, operating theatre and intensive care areas.

Further research is required on the minimum volume of fluid needed to measure IAP via the intra bladder technique, the assessment of the reliability of a single IAP measurement, and a comparison of intra bladder and intra gastric IAP to establish the validity of an alternative route in measuring IAP.

## Conclusion

The pathological characteristics of IAH and ACS have the potential to cause multi organ failure and subsequently increase patient mortality. Monitoring IAP and APP for signs of ACS has become an inexpensive and useful diagnostics tool for identifying complications. An integrated approach to screening and monitoring for IAH may improve patient outcomes and decrease hospital costs. Due to the high incidence of IAH and ACS, it is essential for critical care nurses to regularly monitor IAP and APP. Critical care nurses require advanced clinical practice, skills, knowledge and awareness of the pathological signs, symptoms and complications of IAH and ACS.

### Key points

● Intra-abdominal hypertension (IAH) and abdominal compartment syndrome (ACS) occur frequently in critical care and can alter organ perfusion and end organ function.

● Measurement of Intra-abdominal pressure (IAP) is done via the bladder using the modified Kron technique.

● Abdominal compartment syndrome (ACS) is classified as an IAP greater than 20 mmHg with a new organ dysfunction.

● Critical care nurses play a significant role in the recognition and management of IAH and ACS.

## Competing interests

The authors declare that they have no competing interests.

## Authors’ contributions

LH: Study design, data analysis and interpretation, manuscript preparation. SAF: Study design, interpretation of data, manuscript preparation. KH: Study design, interpretation of data, manuscript preparation. PJN: Interpretation of data, manuscript preparation. PMD: Study design, interpretation of data, manuscript preparation. All authors read and approved the final manuscript.
